# Surgical Treatment of Juvenile Idiopathic Arthritis in the Era of Novel Drug Therapies

**DOI:** 10.3390/jcm12103402

**Published:** 2023-05-11

**Authors:** Céline Klein, Vincent Barbier, Christophe Glorion, Richard Gouron

**Affiliations:** 1 Department of Paediatric Orthopaedics, Amiens Picardie University Hospital, University of Picardie Jules Verne, 80054 Amiens, France; 2 MP3CV-EA7517, CURS—Amiens University Hospital, Jules Verne University of Picardie, 80000 Amiens, France; 3 Paediatric Orthopaedic Surgery Department, Necker University Hospital, Sorbonne Paris Cité, 75015 Paris, France

**Keywords:** juvenile idiopathic arthritis, synovectomy, total hip arthroplasty, total knee arthroplasty, children, soft tissue release

## Abstract

Juvenile idiopathic arthritis is the most common chronic rheumatic disease encountered in children under the age of sixteen and causes significant impairments in daily life. Over the last two decades, the introduction of new drug treatments (including disease-modifying antirheumatic drugs and biologics) has changed the course of this disease, thus reducing the indication for surgery. However, some patients fail to respond to drug therapy and thus require personalized surgical management, e.g., the local reduction of joint effusion or a synovial pannus (via intra-articular corticosteroid injections, synovectomy, or soft tissue release), and management of the sequelae of arthritis (such as growth disorders and joint degeneration). Here, we provide an overview of the surgical indications and outcomes of the following interventions: intra-articular corticosteroid injections, synovectomy, soft tissue release, surgery for growth disorders, and arthroplasty.

## 1. Introduction

Juvenile idiopathic arthritis (JIA) is the most common inflammatory rheumatic condition affecting children under the age of 16, with joint inflammation lasting for more than 6 weeks. The reported incidence of JIA ranges from 1.9 to 23 per 100,000 [1,2], with marked differences in prevalence from one country to another [3]. JIA affects around 300,000 children in the USA [4]. The International League of Associations for Rheumatology distinguishes between seven forms of JIA: the systemic form, the rheumatoid-factor-negative polyarticular form, the rheumatoid-factor-positive polyarticular form, the oligoarticular form, psoriatic arthritis, enthesitis-related arthritis, and undifferentiated arthritis.

The disease mechanisms underlying JIA have not been fully characterized, which makes the condition challenging to manage. The goals of treatment are to: (i) slow down or stop the joint inflammation, (ii) relieve effusion, contracture, and pain, (iii) prevent joint destruction, and (iv) preserve function and quality of life. Over the last two decades, the use of drug treatments (e.g., non-steroidal anti-inflammatories (NSAIDs) and disease-modifying antirheumatic drugs (DMARDs)) has changed the course of JIA, decreased the number of patients experiencing long-term impairments, and thus modified the profile of patients requiring surgery [5,6].

Juvenile idiopathic arthritis should be treated by a multidisciplinary team of pediatric rheumatologists, pediatric orthopedic surgeons, family physicians, and physiotherapists. Children with JIA are frequently first referred to an orthopedic surgeon—especially at the onset of disease, with joint effusion. The initial presentation can mimic septic arthritis, requiring puncture and antibiotic treatment. Disease progression with the recurrence of arthritis confirms the diagnosis of JIA.

The orthopedic surgeon will operate on patients in whom one or more joints fail to respond to drug treatment; otherwise, disease progression will lead to recurrent joint effusion, growth disorders with axial deformities and limb length discrepancy, and early joint destruction. Early management reduces the risk of recurrence and long-term joint degradation [7].

Initially, the symptoms of JIA can be managed in collaboration with the surgical team, with resting, the use of a crutch, and an orthosis. Physiotherapy can help to maintain a joint’s range of motion by encouraging passive and active movement.

After a comprehensive pre-operative assessment, the patient’s follow-up will include ultrasound imaging, X-rays, and MRI with gadolinium contrast enhancement. Musculoskeletal ultrasound is essential for characterizing articular or periarticular arthritis and assessing the treatment response [8].

MRI is useful for assessing the level and extent of synovitis and joint damage [9,10,11,12]. Before endotracheal intubation, X-rays with lateral extension and flexion of the cervical spine should be performed so that the instability of the occipital joint is not underestimated [13].

Several surgical procedures could be indicated in JIA, from intra-articular corticosteroid injection (in cases of persistent joint effusion) to joint replacement (in cases of joint degeneration).

At a time when drug treatment has changed the profile of patients with JIA, this narrative review aims to provide a comprehensive overview of surgical options that have become rare. We focus on five procedures: intra-articular corticosteroid injections, synovectomy, soft tissue release, surgery for growth disorders, and arthroplasty.

The current state of knowledge reviewed here is based on a search of the literature available in PubMed and Google Scholar and published between January 1970 and January 2023. The keywords “juvenile idiopathic arthritis” and “Still’s disease” were combined with “intra-articular corticosteroid injection”, “synovectomy”, “soft tissue release”, “growth disorders”, “arthroplasty”, “total knee arthroplasty”, and “total hip arthroplasty”. All the included articles had to be published in English, and the full text had to be available. We sought to describe the indications, results, and complications of the five surgical procedures mentioned above.

## 2. Intra-Articular Corticosteroid Injection

One of the main surgical options for decreasing inflammation is the intra-articular injection of corticosteroids. According to the American College of Rheumatology guidelines, the intra-articular injection of corticosteroids is recommended for oligoarticular JIA and/or trials of scheduled nonsteroidal anti-inflammatory drugs (NSAIDs) [14]. The intra-articular injection of corticosteroids may also be an adjunct treatment in temporomandibular and polyarthritis forms [7]. Although intra-articular injections are strongly recommended, their incidence in population studies is difficult to define, but appears to range between 20.7% and 48% [15,16]. An optimal age for the first intra-articular injection of corticosteroids has not been reported, although patients receiving their first injection before the age of 7 or as soon as symptoms appeared were in remission for longer [17,18].

This technique is considered safe and effective [19]. It can be performed under local anesthesia, with conscious sedation, or under general anesthesia. Synovial fluid should be aspirated before the corticosteroid injection. The value of splinting and physical therapy after the injection remains subject to debate [20]. Early intra-articular corticosteroid injection is associated with a decrease in joint contracture and greater pain relief and might decrease the risk of limb discrepancy in children with oligo-arthritis [21,22]. With one injection, the joint survival rate was 70% at 1 year and 37% at 3 years, but the rates were lower with 2 or more injections [23].

The two most frequently injected agents are triamcinolone hexacetonide and triamcinolone acetonide, and the former is strongly recommended by the American College of Rheumatology [14]. Remission is longer with triamcinolone hexacetonide than with triamcinolone acetonide, although the most recent results on this topic are contradictory [24,25,26,27,28]. The reported adverse events rates are low, and the most frequent reported adverse event is subcutaneous atrophy [23,29].

## 3. Synovectomy

The main objective of synovectomy is to decrease the volume of intra-articular inflammatory tissue, which is then expected to decrease pain levels, increase joint function, and prevent joint destruction [30]. However, the optimal timing of synovectomy and the expected benefits have not been fully defined [31]. Furthermore, there are no data from randomized, controlled studies of large cohorts with a long follow-up period, and therefore, the role of synovectomy in the treatment of JIA is subject to debate. Trends towards a reduction in pain and an improvement in function were observed in several studies, other than that conducted by Mäenpää et al., in which the range of motion did not increase [31,32,33,34,35] (Table 1). Kvien et al. compared synovectomy (n = 15 patients) with no synovectomy (also n = 15), and the median age at surgery was 136 months [32]. The investigators concluded that synovectomy was associated with significant decreases in pain, joint swelling, and joint disease activity for at least two years [32].

Synovectomy does not prevent joint deterioration [31,33,35]. The risk of recurrence of synovitis greatly varied from one study to another, but was especially high in older children, children with polyarthritis, and children with a highly active disease at the time of the synovectomy [31,36,37,38].

Open synovectomy requires large incisions, which are associated with hematoma and an elevated risk of infection. For the last few decades, arthroscopy has been performed on the wrist, knee, hip, and shoulder joints of patients with JIA [31,36,39,40,41,42,43]. Arthroscopy allows the surgeon to assess the states of the joint’s cartilage before synovectomy and is safe, less invasive, and less morbid than open synovectomy [36,39,40].

Most of the studies of hip synovectomy in JIA are small case series. In contrast, Carl et al. studied 65 hips with a mean follow-up time of 50 months [35,37]. Early active and passive physical therapy was initiated immediately after surgery [35]. The Merle d’Aubigné hip score, pain scores, mobility, and walking ability were significantly better after surgery [35]. The 5-year hip survival rate after total hip arthroplasty (THA) was 87% [35].

Knee synovectomy has been studied more than synovectomy of other joints [30,31,36,39,44]. Arden et al. performed 53 knee synovectomies with a mean follow-up of 4.5 years, and the benefits for pain relief and range of motion were good in the short term but markedly weakened over time [30]. This case series was continued through the inclusion of 76 patients, for whom good results were achieved [45]. Dell’Era et al. retrospectively reviewed 31 arthroscopic knee synovectomies in 19 children (mean age at surgery: 15.6 years) [36]. Two years after surgery, recurrence was observed in 75% of the patients with oligo-arthritis.

Approximately 25% of the patients had synovitis of the elbow [33]. Although elbow synovectomy is rarely performed, it is most frequent among adults with rheumatic arthritis. The elbow appears to respond well to corticosteroid injections. Elbow synovectomy can be combined with radial head excision and anterior capsule release, if needed [33]. Wrist synovectomy can be performed on the ulnar head, radiocarpal joint, and all the intercarpal joints [34].

Post-synovectomy complications are rare, but they include stiffness, wound infections, keloid scarring or hematoma, growth disorders, and thrombophlebitis.

The indication for synovectomy is a joint that does not respond to a well-managed drug treatment, with synovial hyperplasia, persistent effusion after intra-articular corticosteroid injection, and pain. Arthroscopy should be preferred to open synovectomy. Early rehabilitation helps to restore joint range of motion.

## 4. Soft Tissue Release

Surgical soft tissue release can be considered for children with a severe functional impairment. The soft tissue to be released can be adjacent to the hip [46,47], the knee [48,49], or both [50]. Witt systematically combined partial synovectomy with tenotomy [47]. When the objective is to reduce hip contractures, tenotomies of the adductors, psoas muscles, and rectus femoris can be considered [46,47]. For the knee, tenotomy of the hamstrings was possible and could be combined with subperiosteal resection of the gastrocnemius and opening of the capsule [49]. Soft tissue release could also be performed on chronic patella dislocation. Post-operative management included pain relief medication and a combination of physiotherapy and balneotherapy [46,47]. Patients were placed in traction at night or between rehabilitation sessions, for several months [46,47]. In the case of the knee, Rydholm et al. suggested plaster cast immobilization for up to 14 days, followed by use of an orthosis at night for 6 months [49]. Physiotherapy was initiated after surgery and consisted of knee flexion/extension exercises [49].

For the hip, the results were positive in terms of pain reduction, a greater joint range of motion, and better function (e.g., walking in some children who had lost the ability to do so) [46,47,48,50]. The benefits of surgery decreased beyond three years post-operation [48,50]. Soft tissue release does not prevent joint deterioration [46]. Today’s indications for soft tissue release are very limited, and the technique is mainly used as an adjunct to other surgical procedures (e.g., knee replacements).

## 5. Surgery for Growth Disorders

Chronic synovitis and the inflammatory process lead to hyperemia in the area surrounding the growth plate, which can induce accelerated growth, length discrepancy, and axial deviation [51].

Epiphysiodesis (using temporary epiphyseal stapling) has been used to correct knee/ankle valgus deformities and leg length discrepancies [52,53,54,55], but it is only possible in children with an open physis. Skeletal age, predicted growth, and correction potential should be determined, but these estimates remain imprecise in children with JIA. The effect of the stapling should be carefully monitored through regular clinical and radiographic follow-up. Correction during the final growth period reduces the risk of recurrence of the deformity. Before surgery, the valgus deformity varied from 10° to 35° and the length discrepancy varied from 1.5 to 3.5 cm [53,54,55,56]. The mean age at surgery ranged from 6 to 15 years [53,54,55]. Epiphyseal stapling is a safe, easy technique for correcting up to 30 mm in leg length discrepancy and 5° to 20° of valgus deformity [52,53,54,55]. Epiphysiodesis is recommended in patients with knee valgus exceeding 10° and/or a limb discrepancy exceeding 10 mm; again, regular clinical monitoring is required. Eight-figure plates or staples can be used. Physiotherapy can be started immediately after surgery [54].

However, a rebound in the length discrepancy or deformity can be observed after the staples are removed, so a slight hypercorrection is recommended [53]. Complications such as mis-locations, peroneal paralysis, infections, premature physeal plate closure, and varus deformity have been observed after epiphyseal stapling [52,53,54,55,56].

Osteotomy can be performed in skeletally mature patients with a severe knee or ankle deformity.

## 6. Arthroplasty

Arthroplasty has been performed most frequently on hip and knee joints, but has also been described for shoulder, elbow, metacarpophalangeal, and ankle joints [57,58,59,60,61,62]. This operation is indicated in young adults suffering from severe pain and a limited range of motion (due to joint destruction) [63]. Over the last two decades, arthroplasty has become less frequent in patients with JIA under 30 years of age, relative to other indications [64,65]. JIA remains the main indication for THA and for total knee arthroplasty (TKA) in young patients [66]. Polyarticular and systemic forms are associated with a higher risk of joint damage, relative to oligoarticular forms [67,68]. Systemic forms have a lower arthroplasty survival rate and a higher risk of complications, relative to polyarticular forms [67].

Closure of the growth plate during adolescence is not an absolute requirement for arthroplasty [63,69]. It is preferable to first focus on arthroplasty of the upper limbs, impairments of which result in a greater handicap (relative to the lower limbs) in daily life. Next, when considering the lower limbs, the hip is often treated (bilaterally) before the knees [63,70].

Optimal preparation for surgery should take account of the small size of the joint in children (which might require a custom prosthesis), bone stock deficiencies, and any associated joint contracture. The bone stock is less impaired by the use of DMARDs, relative to corticosteroids.

The American College of Rheumatology and the American Association of Hip and Knee Surgeons have issued guidelines on the perioperative management of total hip and knee arthroplasty patients with JIA aged 18 or over [71].

a. Total hip arthroplasty

In cases of JIA, the hip is the joint that most frequently requires arthroplasty [67,72]. THA is an elective treatment for patients who fail to respond to drug therapy and in whom joint degeneration results in pain and a significantly worse quality of life. Kahlenberg et al. evaluated the number of THAs performed in patients under the age of 21 in the USA: the frequency fell from 27% in 2000 to 4% in 2016 [73]. In a cohort of 753 patients (aged 2 to 17) with JIA, hip involvement was observed in 20.6% and THA was required in 2.1% [74]. THA is associated with a reduction in pain and an increase in range of motion [63,75]. Swarup et al. included 56 patients (with 97 primary THAs) between 1982 and 2011 [76]. The hip arthroplasty survival rate was 85% at 10 years and 50% at 20 years, similarly to Marino et al.’s findings [67,76]. At present, the most common bearing surfaces used in young patients are ceramic-on-ceramic and metal-on-crosslinked polyethylene [66,77,78]. The implant survival rate has increased over the last two decades, due to the introduction of highly crosslinked polyethylene, improved surgical techniques, and better perioperative management [77,78].

The 10- to 15-year survival rate for the femoral component of cementless prostheses ranged from 85% to 100% [75,76,79]. However, Malviya et al. found a rate of 46.6% at 19 years, with a trend towards better results for a cemented acetabular component and a cementless femur [72]. Oh reported on the use of a miniature anatomic medullary locking stem in 52 cases, with a mean follow-up period of 7.6 years. The researchers found a post-operative improvement in the Harris hip score and recommended using a cementless femoral stem in patients with small anatomic dimensions [80]. The survival rate was also influenced by long-term use of corticosteroids (a significantly lower rate) or methotrexate (a significantly higher rate) [72].

Patients with JIA receive more transfusions after THA and are exposed to the highest risk of revision for periprosthetic fracture compared to patients with osteoarthritis [81,82,83]. Lastly, patients with JIA are more likely to develop prosthesis infections and sciatic nerve paralysis.

b. Total knee arthroplasty

In JIA, arthritis of the knee leads to pain, a decreased range of motion, and the limitation of activities of daily living, and therefore, may prompt TKA [72,84,85]. TKA in JIA is a challenging procedure. The bone and joint deformities induced by premature closure of the growth plate of the femur and tibia must be taken into account during pre-operative planning and implant selection. Knee stiffness and joint contracture may require a lateral retinaculum release for good surgical exposure [86,87]. Implants can be cruciate-retaining, posterior-stabilized, constrained, hinged, or custom-designed [84]. Patella resurfacing and posterior cruciate ligament resections are options, but they are subject to debate [87]. Simultaneous bilateral TKA can be performed when both knees are involved. Lybäck et al. reviewed 77 knee arthroplasties in JIA patients, with previous synovectomy in 23, epiphyseal stapling in 9, both synovectomy and epiphyseal stapling in 4, and no previous surgery in 24 [44]. The researchers observed that the mean age at arthroplasty was lower in the group with previous surgery [44]. Intensive physiotherapy should be promptly initiated after surgery, in order to optimize the recovery of joint range of motion. In a study of a large cohort of 349 THAs in patients with JIA, Heyse et al. reported a joint survival rate of 95% at 10 years and 82% at 20 years. Marino et al. reported worse 20-year survivorship, with a value of 50% [67]. The complications most frequently observed were aseptic loosening, infections, and stiffness [66,67,70,86,87].

c. Others

Shoulder, elbow, and ankle replacements can also be considered, but are less common than knee and hip replacements. Shoulder arthroplasties reportedly result in improved function, a greater range of motion, and less pain [58,61,62,88]. The choice of the surgical technique is still subject to debate. There are no ideal implants for use in total arthroplasty, stemmed hemiarthroplasty, and resurfacing hemiarthroplasty. Moreover, resurfacing hemiarthroplasty avoids the glenoid complication of total shoulder arthroplasty and preserves the bone stock [86,89].

Elbow arthroplasty is technically challenging and requires implant modification and custom designs [60,89,90,91,92]. Semi-constrained total arthroplasty can be used—especially in cases of stiffness or ankylosis [90]. Even though total elbow arthroplasty improves functional results, complications (such as loosening, instability, and revision) are frequent [89].

## 7. Conclusions

The surgical management of children with JIA requires a multidisciplinary approach. Recent therapeutic approaches with DMARDs are effective and have considerably modified the profile of patients requiring surgery. Intra-articular corticosteroid injection is a safe, effective procedure, and should be considered in cases of persistent joint effusion in cases of the oligo-arthritis form. Considering the absence of recent studies of synovectomies and soft tissue release, the indications for these two surgical operations are rare, and the long-term benefits are subject to debate. For end-stage joint degeneration, arthroplasty is indicated, and can provide significant improvements in function, pain relief, and quality of life.

## Figures and Tables

**Table 1:** Results of synovectomies.

Author	Design	Joint(s)	O/A	N	Age (Years)	Follow-Up (Years)	Complications	Pain	Function	Recurrence
Arden [30]	R	knee	O	53	18	4.5	hemarthroses (3.7%), stiffness (24%)	84% no pain or slight pain	84% excellent or good	Good results at one year but a sharp decrease afterwards
Rydholm [31]	R	knee	O	51	13	7.5	wound infection (n = 2), mobilization under general anesthesia (n = 21)	14 knees at last follow-up		
Kvien [32]	P	wrist/ankle knee	O	30 (15 with synovectomy, 15 without)	9.5–11.3	24 months	keloid scarring (n = 1), knee re-mobilization under general anesthesia (n = 2)	improvement	Improvement, but never as much as patients without synovectomy	
Mäenpään [33]	R	elbow	O	29	29	5	wound infection (n = 1), stiffness (n = 1)	44% with total pain relief	no significant improvement	4 repeat synovectomies, 2 arthroplasties, survival rate of 84% at 5 years
Hanff [34]	R	wrist	O	15	15	3	NA	50% with less pain	increase in grip strength and decrease in range of motion	11 radiographic deterioration, 4 arthrodesis
Carl [35]	R	hip	O	56	13.7	50 months	superficial hematoma (n = 2)	significant improvement	85% with a very great or great improvement	Survival rate of 87%, 5 arthroplasties
Dell’Era [36]	R	knee	A	19	15.6	5.4	Thrombophlebitis (n = 1), septic arthritis (n = 1)	NA		75% for oligoarthritic patients two years after surgery
Jacobsen [37]	R	hip/knee/ ankle/wrist	O	10/22/4/4	11.5	7.1	stiffness (n = 6 knees)	slightly less than before surgery, at last follow-up	no improvement	2 repeat knee synovectomies—arthroplasties (3 knees, 3 hips)
Ovregard [38]	R	hip/knee/ ankle/wrist/ elbow/other	O	212	11.6	3	deformities (n = 9), growth disorders (n = 1), hypertrophic scarring (n = 1)	NA	response after 1–2 years, and then a decrease	
Toledo [39]	R	knee/ shoulder temporomandibular	A	19/1/2	9	57 months	NA	NA	good but not complete range of motion (shoulder)	50% relapse at 24 months
Ishwar [40]	R	wrist	A	15		11.3 months	no complications	median VAS reduction of 4 points	grip strength and functional improvement	

## Data Availability

The data that support the findings of this study are available from the corresponding author, C.K., upon reasonable request.
